# Characterization and *ex vivo* Expansion of Human Placenta-Derived Natural Killer Cells for Cancer Immunotherapy

**DOI:** 10.3389/fimmu.2013.00101

**Published:** 2013-05-01

**Authors:** Lin Kang, Vanessa Voskinarian-Berse, Eric Law, Tiffany Reddin, Mohit Bhatia, Alexandra Hariri, Yuhong Ning, David Dong, Timothy Maguire, Martin Yarmush, Wolfgang Hofgartner, Stewart Abbot, Xiaokui Zhang, Robert Hariri

**Affiliations:** ^1^Celgene Cellular TherapeuticsWarren, NJ, USA; ^2^Princeton UniversityPrinceton, NJ, USA; ^3^Celgene Signal ResearchSan Diego, CA, USA; ^4^Department of Biomedical Engineering, Rutgers UniversityPiscataway, NJ, USA

**Keywords:** placental-derived natural killer cells, *ex vivo* expansion, anti-tumor cytolytic activity, miRNA, cellular immunotherapy

## Abstract

Recent clinical studies suggest that adoptive transfer of donor-derived natural killer (NK) cells may improve clinical outcome in hematological malignancies and some solid tumors by direct anti-tumor effects as well as by reduction of graft versus host disease (GVHD). NK cells have also been shown to enhance transplant engraftment during allogeneic hematopoietic stem cell transplantation (HSCT) for hematological malignancies. The limited *ex vivo* expansion potential of NK cells from peripheral blood (PB) or umbilical cord blood (UCB) has however restricted their therapeutic potential. Here we define methods to efficiently generate NK cells from donor-matched, full-term human placenta perfusate (termed Human Placenta-Derived Stem Cell, HPDSC) and UCB. Following isolation from cryopreserved donor-matched HPDSC and UCB units, CD56+CD3− placenta-derived NK cells, termed pNK cells, were expanded in culture for up to 3 weeks to yield an average of 1.2 billion cells per donor that were >80% CD56+CD3−, comparable to doses previously utilized in clinical applications. *Ex vivo*-expanded pNK cells exhibited a marked increase in anti-tumor cytolytic activity coinciding with the significantly increased expression of NKG2D, NKp46, and NKp44 (*p* < 0.001, *p* < 0.001, and *p* < 0.05, respectively). Strong cytolytic activity was observed against a wide range of tumor cell lines *in vitro*. pNK cells display a distinct microRNA (miRNA) expression profile, immunophenotype, and greater anti-tumor capacity *in vitro* compared to PB NK cells used in recent clinical trials. With further development, pNK may represent a novel and effective cellular immunotherapy for patients with high clinical needs and few other therapeutic options.

## Introduction

The critical role of natural killer (NK) cells in the defense against cancer and virus infection has been increasingly appreciated since they were first discovered in mice more than 30 years ago (Herberman et al., 1975a,b). Clinical studies exploring the biological activities of NK cells in the treatment of malignant disease and during allogeneic hematopoietic stem cell transplantation (HSCT) have provided promising results. Transplant studies have suggested alloreactive NK cells could mediate potent anti-leukemia effects without causing graft versus host disease (GVHD). In human leukocyte antigen (HLA)-mismatched, haploidentical allogeneic stem cell transplants (SCT), NK alloreactivity was associated with a higher rate of survival, a lower rate of relapse, and treatment related mortality post transplantation (Ruggeri et al., 1999, 2002; Velardi et al., 2002). Several clinical studies have convincingly demonstrated that adoptive transfer of NK cells isolated from peripheral blood (PB) of haploidentical donors can be successfully used for immunotherapy in acute myeloid leukemia (AML) patients (Miller et al., 2005; Rubnitz et al., 2010; Curti et al., 2011). However, a number of technical challenges have hampered the widespread application of NK cells in immunotherapy; these include a limited ability to generate large numbers of effector cells, difficulty in maintaining high tumoricidal activity during *ex vivo* expansion and *in vivo* therapy, and a limited understanding of NK-specific tumor targeting profiles. Therefore, there is a need to overcome these challenges and enable a NK cell-based anti-tumor strategy in the clinic.

To date, the most utilized source for NK cells in adoptive immunotherapy is PB (Sutlu and Alici, 2009), with clinically effective doses reported in the range of 1 × 10^6^–9.3 × 10^6^ PB NK cells/kg (Passweg et al., 2004; Miller et al., 2005; McKenna et al., 2007; Shi et al., 2008; Meyer-Monard et al., 2009; Rubnitz et al., 2010; Yoon et al., 2010; Curti et al., 2011). Embryonic stem cells (Woll et al., 2009) and umbilical cord blood (UCB) (Spanholtz et al., 2010) have also been used as sources of CD34+ cells that were differentiated into functional NK cells. Previous studies have highlighted the potential to selectively isolate and expand NK cells from UCB for adoptive cell transfer treatment of tumors (Xing et al., 2010). Over the last decade the phenotype and function of decidual NK (dNK) cells in placenta development have been studied extensively (Koopman et al., 2003; Hiby et al., 2004; Kopcow et al., 2005, 2010; Apps et al., 2011; Male et al., 2011). However, little information is available on the role of NK cells from placenta for cellular immunotherapy.

Recently, human placenta has been demonstrated as a novel and valuable source of multipotential stem/progenitor cells of mesenchymal and hematopoietic origin for multiple therapeutic applications (Parolini et al., 2008; Prather et al., 2008). Celgene Cellular Therapeutics (CCT, a division of Celgene Corporation) is developing human placenta-derived stem cells (HPDSC) as an adjunct to UCB cells for allogeneic use in first-degree or second-degree blood relatives for augmentation of the stem cell graft in hematopoietic reconstitution. We have established a standardized procedure to perfuse donated full-term placentas with normal saline to recover HPDSC. HPDSC were subsequently processed to remove red blood cells, non-viable cells and tissue debris followed by cryopreservation. HPDSC were neither expanded nor cultured during processing. The process typically yields 100–500 million total nucleated cells (TNC), approximately 1–5% of which are CD34+ hematopoietic stem cells (HSCs). We hypothesize that HPDSC combined with the donor-matched UCB could represent an effective new source of NK cells that holds potential for further immunotherapeutic development.

Unlike their antigen-specific lymphoid counterparts, such as T cells and B cells, NK cells, characterized as CD56+CD3−, recognize and subsequently kill virus-infected and transformed cells without prior immunization. NK cells operate via the balance of signals from inhibitory receptors, such as the killer cell immunoglobulin-like receptors (KIRs), and the C-type lectin family receptor: CD94/NKG2, with activating receptors, such as NKG2D, NKp46, NKp44, NKp30, and CD226 (Smyth et al., 2002; Huntington et al., 2007). Two major subtypes of CD56+ NK cells can be distinguished according to the co-expression of the cell surface marker CD16 (Jacobs et al., 2001). It has been demonstrated that CD56+CD16− NK cells have very few cytolytic granules, low or no expression of KIRs, high expression of KLR family members and are capable of producing cytokines and chemokines upon activation. CD56+CD16+ NK cells have abundant cytolytic granules and high expression of KIRs. PB contains more than 90% CD56+CD16+ NK cells, while more than 90% of NK cells in lymph nodes do not express CD16 (Cooper et al., 2001; Fehniger et al., 2003). Results from developmental NK cells studies suggest that the CD56+CD16+ NK cells are derived from the CD56+CD16− NK cells (Lanier et al., 1986; Ferlazzo et al., 2004; Freud et al., 2005). In addition to cell surface markers, different miRNA expression profiles have been associated with NK cell development, maturation, and function (Bezman et al., 2010). To date, no study has investigated the miRNA profile starting from donor-matched HPDSC and UCB (hereafter referred to as “Combo unit”) followed by differentiation into functional NK cells.

In this study, we report that placenta is a rich source of placenta-derived NK (pNK) cells that can be readily isolated from Combo units, followed by *ex vivo* expansion. We evaluated the proliferation, immunophenotype, miRNA expression, and activation of these expanded cells, as well as their cytolytic activities *in vitro*. Our results demonstrate that pNK cells can be generated in clinically relevant quantities and may be developed as a highly cytotoxic cellular product that can be used to treat a wide range of cancers.

## Materials and Methods

### Processing of HPDSC and UCB

Postpartum placentas and umbilical cords were procured under full-informed consent of donors with donor eligibility documentation, and were qualified using a series of tests, including serology, bacteriology, and HLA typing. HPDSC isolation and recovery was achieved by cannulation of the umbilical vessels (two arteries and one vein) under sterile conditions with polyethylene catheters connected to a flow-controlled fluid circuit allowing perfusion of the placenta. A total of 750 ml of perfusion solution (0.9% NaCl injection solution USP Grade) (VWR) was collected from each placenta. UCB was obtained by cannulation of the umbilical vein and collected into a bag containing citrate-phosphate-dextrose (Fenwal). Both UCB and perfusate were then processed by red blood cell depletion using Hetastarch, followed by volume reduction. The resulting cell populations were cryopreserved in a solution containing 5% human albumin and 10% DMSO with a controlled rate freezer prior to final storage in the gas phase of a liquid nitrogen tank.

### Isolation of pNK cells from HPDSC and UCB

The donor-matched cryopreserved HPDSC and UCB were initially thawed, combined, and washed with RPMI 1640 (without phenol red) (Gibco) containing 5% v/v fetal bovine serum (FBS; Hyclone Laboratories). In some NK cell expansion studies, peripheral blood mononuclear cells (PBMCs) obtained from buffy coat (Blood Center, NJ, USA) were prepared as an alternate source of NK cells. After washing, cell pellets were resuspended at 5 × 10^7^ cells/ml in RoboSep buffer (StemCell Technologies). DNase I (0.1 mg/ml solution) (StemCell Technologies) was added to the cell suspension to a final concentration of 100 μl/ml, mixed gently by pipetting and incubated for 15 min at room temperature (RT) prior to isolation of NK cells using the EasySep^®^ NK Cell Enrichment Kit (StemCell Technologies). Human NK Cell Enrichment Cocktail (containing monoclonal antibodies to human cell surface antigens CD3, CD4, CD14, CD19, CD20, CD36, CD66b, CD123, HLA-DR, and glycophorin A) was added to the cell suspension at a final concentration of 50 μl/ml and incubated for 10 min at RT. After incubation with EasySep^®^ Magnetic Microparticles (final concentration of 100 μl/ml) for 5 min at RT, enrichment of NK cells was performed according to the protocol provided by the manufacturer (StemCell Technologies). A CD56+CD3− population was thus collected and ready for further analysis or cultivation. The following equation was used for the calculation of the recovery of pNK cells: (purified NK cell count × purified CD56+CD3−%)/(TNC count × starting CD56+CD3−%).

### *Ex vivo* expansion of pNK cells

Enriched placental CD56+CD3− NK cells were cultured in Start Medium based on a modification of previously described protocols (Yssel et al., 1984). All components were from Sigma-Aldrich unless otherwise specified. Briefly, Start Medium was composed of Iscove’s Modified Dulbecco’s Media (IMDM) (ATCC) supplemented with 10% FBS (Hyclone), 35 mg/ml transferrin, 5 μg/ml insulin, 20 μM ethanolamine, 1 μg/ml oleic acid, 1 μg/ml linoleic acid, 0.2 μg/ml palmitic acid, 2.5 μg/ml bovine serum albumin (BSA), and 0.1 μg/ml Phytohemagglutinin (PHA-P). NK cells were resuspended at approximately 2.5 × 10^5^/ml in Start Medium plus Penicillin-Streptomycin (Invitrogen) and 200 IU/ml IL-2 (R&D Systems). Mitomycin C-treated PBMC and K562 (ATCC) cells were added together to Start Medium as feeder cells at a final concentration of 1 × 10^6^/ml each. To initiate NK cell expansion, the feeder cells and NK cell suspension was transferred into a gas permeable culture bag (American Fluoroseal) and was cultured in an incubator at 37°C in 5% CO_2_. After culturing for 5–7 days, expanded cell populations were fed with Maintenance Medium for up to 21 days. Maintenance Medium was composed of IMDM supplemented with 10% FBS, 2% Human AB serum (Gemini), Penicillin-Streptomycin, and 200 IU/ml IL-2. Total cell number and cell viability were assessed using EasyCount (Immunicon) and EasyCount ViaSure Kit (Immunicon). Fold expansion was calculated using the absolute number of CD56+CD3− NK cells on Day 21/absolute number of NK cells on Day 0.

### BRDU/7-AAD cell cycle analysis

Expanded cells at different time points as indicated were labeled with 5-bromo-2′-deoxyuridine (BrdU) (BD Bioscience) and cultured at 37°C in 5% CO_2_ for 24 h. The cells were harvested, fixed, and stained with anti-BrdU and 7-aminoactinomycin-D (7-AAD) following the protocol provided by the manufacturer. The cell cycle data was collected via FACSCalibur (BD Biosciences), and analysis was accomplished with FlowJo (Tree Star, Inc.).

### Immunophenotypic characterization

The phenotype of mononuclear cells (MNCs) or enriched NK cells from Combo unit, or expanded cells from Day 7, 14, 21 cultures, was analyzed by multi-color flow cytometry. Cells were stained with fluorochrome-conjugated monoclonal antibodies against human blood surface antigens: CD56-PerCP/-PE/-PE-Cy7, CD3-FITC/-APC-Cy7, CD16-FITC/-PerCP, CD158b-PE (KIR2DL2/2DL3), CD158e1-PE (KIR3DL1), NKG2D-APC, NKp46-APC, NKp44-PE, NKp30-PE, CD226-PE, 2B4-PE (all purchased from BD Biosciences Pharmingen), and CD94-PE (R&D Systems). All analyses were performed using FACSCanto I (BD Biosciences) and FlowJo analysis software.

### PKH26/TO-PRO-3 cytotoxicity assay

Natural killer cell *in vitro* cytotoxicity was examined using NK cells as effector cells and various tumor cell lines as target cells. Target cells were labeled with PKH26 (Sigma-Aldrich) (Lee-MacAry et al., 2001; Ferlazzo et al., 2004), placed in 96-well U-bottom tissue culture plates and incubated with effector cells at various effector to target (E:T) ratios in 200 μl RPMI 1640 supplemented with 10% FBS. After 4 h incubation at 37°C in 5% CO_2_, cells were harvested and TO-PRO-3 (Invitrogen) was added to cultures at 1 μM final concentration followed by FACS analysis using BD FACSCanto I. Cytotoxicity was expressed as the percentage of dead cells (PKH26+TO-PRO-3+) within the total PKH26+ target tumor cells.

### Lactate dehydrogenase release assay

Alternatively, NK cell *in vitro* cytotoxicity was examined by lactate dehydrogenase (LDH) release assay using CYTOTOX 96^®^ colorimetric cytotoxicity assay kit (Promega). In this assay, effector cells and target cells were placed in 96-well U-bottom tissue culture plates and incubated at various E:T ratios in 100 μl RPMI 1640 without phenol red (Invitrogen) supplemented with 2% human AB serum and incubated for 4 h at 37°C in 5% CO_2_. After incubation, 50 μl supernatant was transferred to the enzymatic assay plate for detection of LDH activity as instructed by the manufacturer. Cytotoxicity was calculated using the following equation: % Cytotoxicity = (experimental release – effector spontaneous release – target spontaneous release)/(target maximum release – target spontaneous release) × 100.

### miRNA preparation and quantitative PCR

MicroRNA was isolated from 0.5 to 1.5 × 10^6^ cells using a MIRVANA™ miRNA Isolation Kit (Ambion) following the protocol provided by manufacturer. The concentration and purity of the recovered small RNA was determined by measuring its absorbance at 260 and 280 nm. Purified RNA samples were subjected to cDNA synthesis using TAQMAN^®^ Reverse Transcription Reagents (Applied Biosystems) followed by real-time PCR analysis by the 7900HT Fast Real-Time PCR System. Human miRNA Arrays (Applied Biosystems) were used for gene expression profiling and miRNA profiling. For each miRNA, the mean ΔCt from real-time PCR was calculated as ΔCt_mean_ = mean(Ct_sample_) – mean(Ct_endo_), where Ct_sample_ is the Ct value of a miRNA and Ct_endo_ is the Ct value of the endogenous control. miRNAs were unique to pNK or PB NK if they met the following criteria: (i) ΔCt_mean_ < 0, (ii) |ΔCt_mean_| ≥ 2 × Ct_endo_SD_, where |ΔCt_mean_| is the absolute value of ΔCt_mean_ and Ct_endo_SD_ is the standard deviation of the Ct_endo_, and (iii) the miRNAs that satisfied the previous two criteria were exclusive to the cell type. The rationale for using the aforementioned criteria was to confirm that a miRNA is abundant in at least one donor sample, in comparison to the endogenous control. Donor samples without detectable levels of a miRNA were numerically ignored when averaging the Ct values. A negative ΔCt_mean_ along with two standard deviations from the control gene ensures that the particular gene is relatively abundant. Additionally, all of the standard deviations of the reference genes were less than 0.25, which confirms the quality of the control. Significantly expressed miRNAs between pNK and PB NK, as well those between Day 0 pNK and Day 21 pNK were determined by a two-sample *t*-test (*p* value of 0.01 significance level) on the ΔCt values. Expression fold changes were calculated according to *R* = 2^(ΔCt1_mean−ΔCt2_mean)^ where *R* is the fold change (Livak and Schmittgen, 2001); ΔCt1_mean is the average ΔCt of pNK on Day 0, and ΔCt2_mean is the average ΔCt of PB NK or expanded pNK.

### miRNA target prediction and pathway analysis

A search for miRNAs that were unique to either PB NK and pNK, as well as those that were highly expressed in expanded pNK, was conducted within seven miRNA target gene prediction databases (Diana-microT, miRDB, miRTar, microRNA.org, MicroCosmTargets, picTar, and TargetScan) and three experimentally validated target gene databases (TarBase, miRecords, and miRTarBase) by using medium to high stringency search criteria. Genes that were predicted by five or more databases were considered as high confidence targets. Such targeted genes were then examined in pathway analysis (Ingenuity Systems) in order to determine the associated signaling pathways and cellular functions. Pathways were scored and ranked based on their *p*-values.

## Results

### Phenotypic profile of HPDSC NK cells

**Figure 1:**
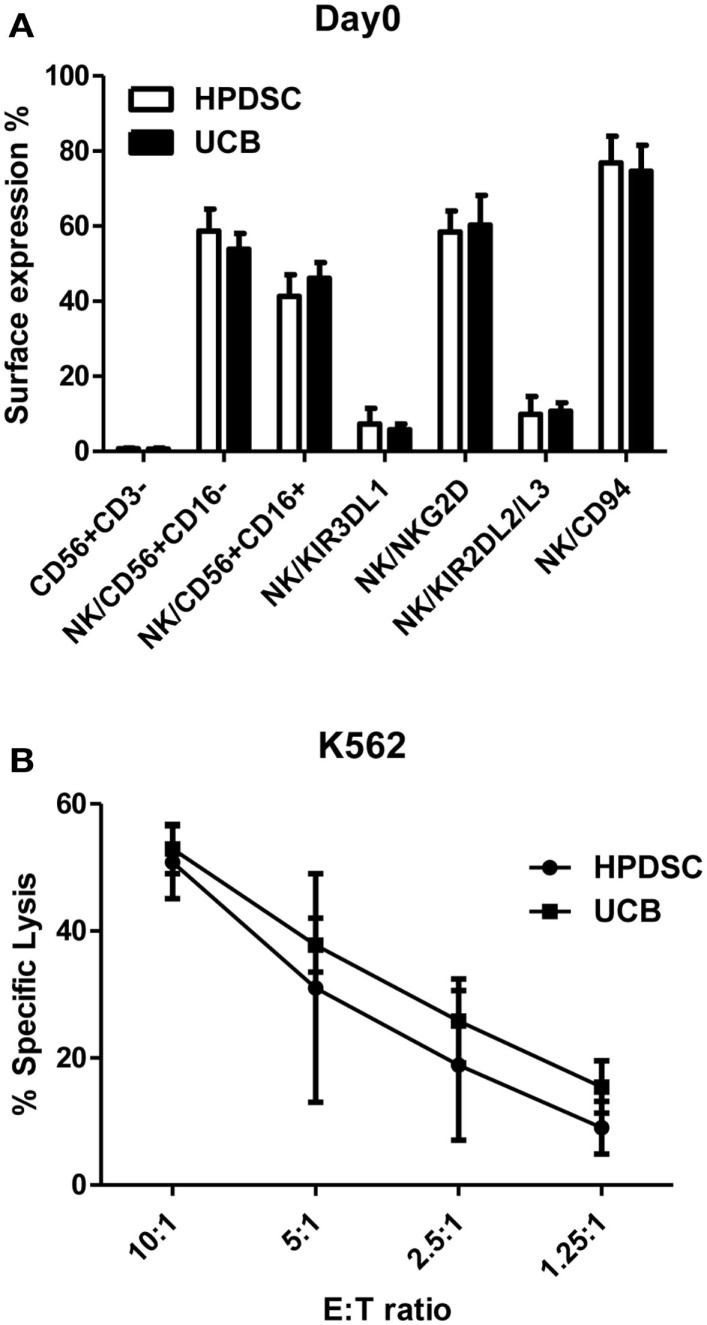
**Comparison of NK cells from donor-matched HPDSC and UCB units**. **(A)** Immunophenotypic characterization of NK cells from donor-matched HPDSC and UCB units, the two-sample *t*-test was used to determine if population means are equal in HPDSC and UCB. **(B)** Cytotoxicity of expanded NK cells from donor-matched HPDSC and UCB units against K562 cells.

Human placenta-derived stem cells was harvested from three placentas separately and analyzed for cell surface markers by flow cytometry and compared to the donor-matched UCB. The composition of NK cells identified by CD56+CD3− expression was not substantially different between HPDSC (0.70 ± 0.24%) and the donor-matched UCB (0.63 ± 0.36%) (*n* = 3). The CD56+CD3− NK cells were then examined in greater detail using fluorescence-conjugated monoclonal antibodies against specific NK receptors. The two-sample *t*-test was used to determine if population means were equal in HPDSC and UCB. As shown in Figure 1A, NK cells from three pairs of donor-matched HPDSC and UCB units exhibited phenotypic similarities, with no significant differences in expression of sub-populations such as CD56+CD16−, CD56+CD16+, NKG2D, CD94, KIR3DL1, and KIR2DL2/L3. After NK expansion for 21 days separately, HPDSC NK, and UCB NK cells showed comparable cytotoxicity against K562 cells at various E:T ratios, indicating functional similarity between HPDSC and UCB NK cells after expansion (Figure 1B).

Based on these similar phenotypic and functional characteristics and in order to increase the starting cell number, HPDSC and donor-matched UCB units were combined into one Combo unit, to be used as the starting material for further cell expansion.

### Isolation and characterization of pNK from cryopreserved combo units

**Figure 2:**
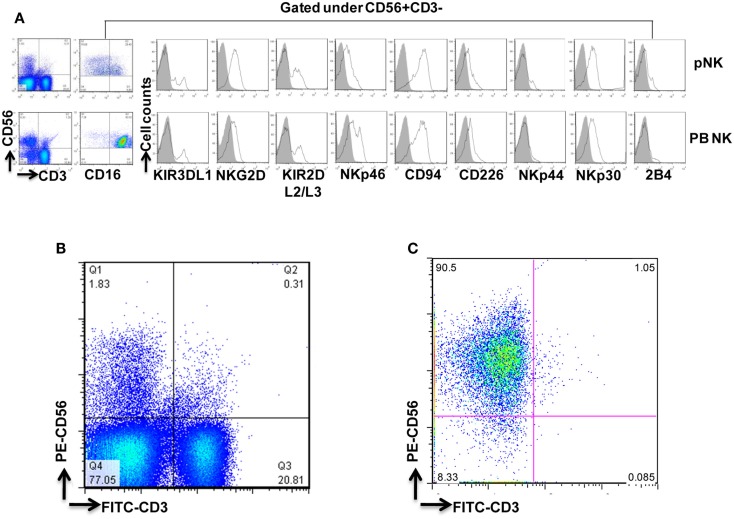
**Phenotypic characterization of pNK cells in comparison with PB NK cells**. **(A)** Flow cytometric identification of NK cells from Combo unit and PB. CD56+CD3− gated NK cells expressed a repertoire of receptors important for regulating NK-cell activity, including CD16, KIR3DL1, NKG2D, KIR2DL2/L3, NKp46, CD94, CD226, NKp44, NKp30, and 2B4. **(B)** Percentage of CD56+CD3− pNK cells from Combo unit prior to NK cell isolation. **(C)** About 90% CD56+CD3− NK cells from Combo unit was achieved after NK cell isolation.

To qualify pNK as a reliable feed stock for this study and for future clinical production, a series of NK cell isolation experiments were performed to evaluate the consistency of recovery of pNK cells from the cryopreserved Combo units. Among 30 isolation procedures performed, an average number of 1.5 × 10^7^ pNK cells were recovered, enriching the abundance of CD56+ CD3− cells approximately 25-fold (71% compared to 3% in the starting material) (Table 1; Figures 2B,C). Our results indicated nearly 90% recovery of pNK cells from the cryopreserved Combo units.

**Table 1:** **NK cell isolation from 30 cryopreserved Combo units**.

	Starting TNC count	Purified NK cell count	Purity (% CD56+CD3)	
			Starting	Purified
Range (min, max)	4.90E + 07, 9.76E + 08	3.97E + 05, 5.90E + 07	0.56, 13.9	23.1, 89.1
Median	3.80E + 08	1.02E + 07	2.40	79.80
Average	3.95E + 08	1.46E + 07	2.88	71.18
SD	2.46E + 08	1.36E + 07	2.63	20.86

*SD, standard deviation*.

To compare pNK cells to PB NK cells, pNK cells from 16 Combo units and NK cells from 13 units of buffy coat obtained from PB were subjected to an extensive immunophenotypic characterization. The expression of cell surface markers, including KIRs (KIR3DL1, KIR2DL2/3), CD94, NKG2D, natural cytotoxicity receptors NCRs (NKp46, NKp44, and NKp30), 2B4, and CD226 was evaluated. Significant differences were observed in 7 out of 11 sub-populations, including CD56+CD16−, CD56+CD16+, KIR2DL2/3+, NKp46+, NKp30+, 2B4+, and CD94+ (Figure 2A; Table 2). Notably, most (79%) of the PB NK cells displayed the mature CD56+CD16+ phenotype, while a majority of the pNK population (>60%) were immature CD56+CD16− cells.

**Table 2:** **Comparison of sub-populations within pNK and PB NK cell phenotype**.

NK sub-populations	Combo (*n* = 16 U)		PB (*n* = 13 U)		*p* Value
	Mean (%)	SD	Mean%	SD	
CD16−	60.94	16.58	21.38	14.00	***
CD16+	39.05	16.58	78.63	14.01	***
KIR3DL1+	12.31	8.11	7.07	8.28	NS
KIR2DL2/L3+	21.89	8.65	9.46	11.31	**
NKG2D+	42.11	17.79	29.88	22.64	NS
NKp46+	6.98	4.33	18.86	13.97	*
CD226+	15.97	6.66	26.75	23.31	NS
NKp44+	9.48	5.27	4.89	6.40	NS
NKp30+	39.08	19.06	18.99	20.86	**
2B4+	11.07	5.90	4.46	6.45	*
CD94+	71.31	13.94	26.17	30.49	***

NS, not significant; *p < 0.05; **p < 0.01; ***p < 0.001.

In attempts to compare gene expression profiles in pNK cells to PB NK cells, we discovered that individual donor variations in gene expression profiles were too large to evaluate differences between the placenta and PB NK sources. As an alternative strategy, differences were further explored by miRNA analysis using TaqMan Array Human MicroRNA Cards to compare expression of 365 miRNAs in pNK and PB NK cells. These analyses identified four miRNAs unique to pNK cells (has-miR-337, has-miR-422a, has-miR-549, and has-miR-618) and eight miRNAs uniquely expressed by PB NK cells (has-let-7b, has-miR-146b, has-miR-19b, has-miR-24, has-miR-347, has-miR-381, has-miR-517c, and has-miR-631). The pNK-unique miRNAs have not been well characterized except hsa-miR-337, which has been associated with chondrogenesis as a regulator of TGFBR2 expression (Zhong et al., 2012). Additionally, 20 miRNAs were expressed at a significantly higher level, and 29 miRNAs were expressed at a significantly lower level in pNK cells compared to PB NK cells (Tables 3 and 4). Target gene prediction analysis returned 14 highly expressed miRNAs in pNK and 24 highly expressed miRNA in PB NK with more than one target gene (Table 5). Thus PB NK cells and pNK cells display distinct miRNA expression patterns.

**Table 3:** **Highly expressed miRNAs in pNK cells**.

miRNA	Fold increase in pNK	*p* Value
hsa-miR-211	5.26	6.73E−03
hsa-miR-520c	5.58	7.70E−03
hsa-miR-125b	7.46	9.26E−04
hsa-miR-100	11.19	4.29E−04
hsa-miR-326	14.50	5.05E−05
hsa-miR-519c	18.74	6.32E−03
hsa-miR-515-5p	20.99	6.88E−03
hsa-miR-450	21.31	3.00E−03
hsa-miR-198	27.97	3.41E−04
hsa-miR-522	33.63	2.56E−03
hsa-miR-518e	39.78	7.17E−03
hsa-miR-497	54.47	8.88E−03
hsa-miR-566	75.98	1.32E−04
hsa-miR-519d	96.65	3.47E−04
hsa-miR-627	98.54	3.42E−04
hsa-miR-524	106.36	7.97E−04
hsa-miR-520g	291.10	3.24E−04
hsa-miR-302c	396.53	3.55E−04
hsa-miR-512-3p	640.56	3.16E−05
hsa-miR-520h	1793.82	9.60E−05

**Table 4:** **Highly expressed miRNAs in PB NK cells**.

miRNA	Fold increase in PB NK	*p* Value
hsa-miR-331	1.43	5.33E−03
hsa-miR-186	1.90	4.54E−03
hsa-miR-17-5p	2.38	2.34E−03
hsa-miR-26a	2.66	2.36E−03
hsa-miR-133b	2.69	8.19E−04
hsa-miR-181b	2.77	4.42E−03
hsa-miR-222	2.83	5.76E−03
hsa-miR-197	3.00	5.48E−05
hsa-miR-146b	3.05	2.92E−03
hsa-miR-342	3.06	3.23E−04
hsa-miR-181d	3.08	3.41E−03
hsa-miR-155	3.12	8.24E−04
hsa-miR-484	3.18	1.23E−03
hsa-let-7g	3.18	2.08E−03
hsa-miR-200c	3.66	1.91E−03
hsa-miR-181c	3.83	2.72E−04
hsa-miR-191	4.06	3.16E−04
hsa-miR-596	4.14	7.06E−03
hsa-miR-142-5p	4.63	4.84E−04
hsa-miR-95	4.86	2.99E−03
hsa-let-7a	5.04	3.91E−04
hsa-miR-21	5.10	2.87E−04
hsa-miR-152	5.46	1.76E−03
hsa-miR-642	5.56	4.70E−04
hsa-miR-24	5.91	2.54E−05
hsa-miR-10a	14.56	5.71E−03
hsa-miR-429	31.74	5.70E−03
hsa-let-7b	108.34	4.66E−05
hsa-miR-199b	2819.55	3.05E−03

**Table 5:** **Differentially regulated miRs and their validated target genes in pNK, compared to PB NK**.

miR	Experimentally validated genes					
**HIGHLY EXPRESSED MIRNAS IN PB NK**						
hsa-let-7a	TRIM71 (7)	HMGA1 (4)	LIN28A (3)	ACP1 (2)	E2F2 (2)	SMOX (1)
	HMGA2 (6)	THBS1 (4)	CASP3 (3)	RTCD1 (2)	ITGB3 (2)	MYC (1)
	UHRF2 (5)	NRAS (3)	PRDM1 (2)	CCND2 (2)	LIN28 (1)	TUSC2 (1)
	MED28 (4)	EIF2C4 (3)	DICER1 (2)	SLC20A1 (2)	NF2 (1)	BCL2 (1)
	ZFP36L1 (1)	NKIRAS2 (1)	EGR3 (1)	IL6 (1)	NEFM (1)	
hsa-let-7b*	HMGA2 (6)	CDC25A (3)	GRPEL2 (2)	NXT2 (2)	CDIPT (1)	SLC25A13 (1)
	IGF2BP1 (5)	AURKB (3)	MARS2 (2)	EIF2C3 (2)	CDKAL1 (1)	SLC25A1 (1)
	TMEM2 (5)	DHX57 (3)	MRM1 (2)	CCNA2 (2)	CSNK1D (1)	UHRF1 (1)
	LIN28B (5)	FNDC3A (3)	POM121 (2)	CCNF (2)	DOCK5 (1)	C20ORF72 (1)
	CCNJ (5)	RDH10 (3)	PXDN (2)	EDEM3 (2)	FADS2 (1)	SCAMP3 (1)
	CDC34 (5)	SLC25A24 (3)	SCYL1 (2)	TRABD (2)	FAM96A (1)	C2ORF18 (1)
	IGF2BP2 (5)	SNAP23 (3)	SLC25A32 (2)	PLAGL2 (2)	GPR56 (1)	CIAO1 (1)
	DMD (5)	LIN28A (3)	SPRYD4 (2)	FARP1 (2)	IPO4 (1)	BIRC6 (1)
	E2F6 (5)	PRDM1 (3)	TAF9B (2)	C7ORF58 (2)	KIAA0409 (1)	AURKA (1)
	HMGA1 (4)	NRAS (3)	TTC9C (2)	LIN28 (1)	NEDD4 (1)	ALG3 (1)
	PGRMC1 (4)	RRM2 (3)	DLC1 (2)	BCL7A (1)	OPRS1 (1)	ARID3A (1)
	THBS1 (4)	CCND1 (2)	CCND2 (2)	ACTG1 (1)	RHOB (1)	CCBL2 (1)
	PDE12 (4)	ATP6V1F (2)	DICER1 (2)	AARSD1 (1)	RHOG (1)	RRP1B (1)
	E2F5 (4)	GEMIN7 (2)	GTF2I (2)	ANAPC1 (1)	SLC1A4 (1)	TAB2 (1)
hsa-let-7g	HMGA2 (6)	EIF4G2 (5)	IGF2BP1 (5)	COL1A2 (3)	BCL2L1 (1)	
hsa-miR-10a	NCOR2 (5)	MAP3K7 (4)	HOXA1 (1)	USF2 (1)	BTRC (1)	
hsa-miR-133b	BCL2L2 (3)	MCL1 (2)				
hsa-miR-146b*	TRAF6 (7)	IRAK1 (5)	MMP16 (2)	CARD10 (1)		
hsa-miR-152	DNMT1 (5)	HLA-G (2)				
hsa-miR-155	SOCS1 (5)	DET1 (4)	SPI1 (3)	SMAD1 (2)	TM6SF1 (1)	RAB34 (1)
	TSHZ3 (5)	IKBKE (3)	MEIS1 (2)	TRAM1 (2)	FOXO3 (1)	RAB6A (1)
	HIVEP2 (5)	PHF17 (3)	SMAD2 (2)	TRIP13 (2)	ARL5B (1)	SYPL1 (1)
	TAB2 (4)	BACH1 (3)	CYR61 (2)	FGF7 (2)	ATG3 (1)	VAMP3 (1)
	JARID2 (4)	RCN2 (3)	TP53INP1 (2)	KRAS (2)	ATP6V1C1 (1)	WDFY1 (1)
	CEBPB (4)	RCOR1 (3)	ANKFY1 (2)	HIF1A (2)	BET1 (1)	ETS1 (1)
	ARID2 (4)	ZNF652 (3)	CHAF1A (2)	C5ORF41 (2)	CBFB (1)	INPP5D (1)
	DHX40 (4)	MYB (3)	CLDN1 (2)	IKBIP (2)	DNAJB1 (1)	PAPOLA (1)
	PICALM (4)	TLE4 (3)	MYO10 (2)	TWF1 (2)	DSG2 (1)	SERTAD2 (1)
	TRIM32 (4)	CSF1R (3)	NARS (2)	CUX1 (2)	FMNL2 (1)	ERMP1 (1)
	ZIC3 (4)	FAR1 (3)	PHC2 (2)	SLA (2)	PKN2 (1)	C3ORF58 (1)
	KBTBD2 (4)	ZNF236 (3)	SDCBP (2)	AGTR1 (1)	PRAF2 (1)	HNRNPA3P1 (1)
	MSH2 (1)	PELI1 (1)				
hsa-miR-17-5p	E2F1 (1)					
hsa-miR-181b	GRIA2 (4)	GATA6 (4)	TIMP3 (4)	MAP3K10 (3)	NLK (2)	PLAG1 (2)
CYLD (2)	VSNL1 (1)	KAT2B (1)	BCL2 (1)			
hsa-miR-181c	GATA6 (4)	KRAS (3)	NLK (2)	NOTCH4 (2)	NOTCH2 (1)	
hsa-miR-181d	GATA6 (3)	NLK (2)	BCL2 (1)			
hsa-miR-186	AKAP12 (1)					
hsa-miR-191	TMC7 (2)	SOX4 (1)				
hsa-miR-197	FBXW7 (4)	DPH1 (2)	UMPS (2)	CLIC1 (1)	HNF4A (1)	FOXO3 (1)
	CHIC2 (4)	ALMS1 (2)	CPNE6 (2)	WDR6 (1)	PEX13 (1)	IL1R1 (1)
	ACVR1 (4)	CES1 (2)	RBM4 (2)	NEK4 (1)	C1ORF38 (1)	CPSF1 (1)
	RAB28 (3)	ZNF302 (2)	CYLD (2)	PIPOX (1)	MED16 (1)	SNX1 (1)
	HNRNPD (2)	RAD51 (2)	RFX1 (2)	IGF2AS (1)	LRP4 (1)	KLF10 (1)
	GOLGB1 (2)	RXRB (2)	IER3 (1)	DCBLD2 (1)	TSPYL1 (1)	AGR2 (1)
	TUSC2 (1)	EHD2 (1)				
hsa-miR-199b	LAMC2 (1)	HES1 (1)				
hsa-miR-200c	ZFPM2 (6)	ZEB1 (5)	ERRFI1 (5)	ZEB2 (5)	FN1 (5)	UBE2I (3)
	BAP1 (2)	PTPN13 (1)	BMI1 (1)	JAG1 (1)	TUBB3 (1)	
hsa-miR-21	TGFBI (5)	GLCCI1 (3)	RASGRP1 (3)	SGK3 (2)	ANKRD46 (2)	SLC16A10 (1)
	NFIB (4)	SOX5 (3)	MSH2 (3)	RP2 (2)	ACTA2 (1)	TIMP3 (1)
	RECK (4)	JAG1 (3)	PCBP1 (3)	SERPINB5 (2)	BTG2 (1)	TGFBR2 (1)
	PDCD4 (3)	BMPR2 (3)	TOPORS (3)	SPRY2 (2)	SESN1 (1)	NCAPG (1)
	FAM3C (3)	TIAM1 (3)	APAF1 (2)	RHOB (2)	SOCS5 (1)	IL1B (1)
	RTN4 (1)	PTX3 (1)	CDK2AP1 (1)			
hsa-miR-222	CDKN1B (5)	FOS (5)	KIT (4)	CDKN1C (3)	PPP2R2A (2)	MMP1 (1)
	SOD2 (1)	BBC3 (1)	PTEN (1)	ICAM1 (1)	ESR1 (1)	
hsa-miR-24*	CDKN1B (4)	TRIB3 (4)	MAPK14 (2)	FURIN (2)	BRCA1 (1)	NOTCH1 (1)
	ACVR1B (4)	DND1 (3)	NFAT5 (2)	MLEC (2)	KIAA0152 (1)	CDKN2A (1)
	KHSRP (1)	HNF4A (1)	TGFB1 (1)			
hsa-miR-26a	SMAD1 (5)	HMGA1 (5)	CDK8 (4)	HMGA2 (3)	CCND2 (2)	CCNE2 (2)
	STRADB (5)	GSK3B (5)	MTDH (4)	CPEB4 (3)	CTGF (2)	LIF (2)
	PTEN (5)	EZH2 (4)	MAP3K2 (4)	SERBP1 (2)	CDC6 (2)	CPEB2 (1)
	CPEB3 (1)	SMAD4 (1)				
hsa-miR-331	ERBB2 (2)	CDCA5 (2)	KIF23 (1)			
hsa-miR-342	BMP7 (1)	GEMIN4 (1)				
hsa-miR-429	ZFPM2 (6)	ZEB1 (6)	ERRFI1 (4)	ZEB2 (4)	WASF3 (3)	BAP1 (1)
**HIGHLY EXPRESSED MIRNAS IN PNK**						
hsa-miR-100	MTOR (3)	PLK1 (1)	FGFR3 (1)	IGF1R (1)	ATM (1)	
hsa-miR-125b	LACTB (6)	BMF (3)	SAMD10 (3)	MKNK2 (2)	BBC3 (2)	DICER1 (1)
	BAK1 (5)	BMPR1B (3)	EIF4EBP1 (3)	CBFB (2)	QSOX2 (2)	JUB (1)
	ARID3B (5)	ENTPD4 (3)	KLF13 (3)	LIN28A (2)	LIN28 (1)	DDX19B (1)
	IRF4 (5)	TOR2A (3)	ULK3 (3)	IGF2 (2)	C10ORF104 (1)	PABPC1 (1)
	PRDM1 (4)	KCNS3 (3)	SLC7A6 (3)	NKIRAS2 (2)	B3GALT4 (1)	AKT1 (1)
	SLC35A4 (4)	LIN28B (3)	SLC7A1 (3)	SEL1L (2)	UBE2I (1)	TP53 (1)
	CGN (4)	GRIN2A (3)	ERBB3 (2)	ATXN1 (2)	RBM8A (1)	CASC3 (1)
	PPAT (4)	STAT3 (3)	CDKN2A (2)	RAF1 (2)	IGFBP3 (1)	E2F3 (1)
	CBX7 (3)	LIF (3)	ABTB1 (2)	CYP24A1 (2)	MAN1A1 (1)	RNF144A (1)
	SGPL1 (3)	SMARCD2 (3)	ARID3A (2)	ABCC4 (2)	SMO (1)	LYPLA2 (1)
	PLEKHA8 (1)	TP53INP1 (1)	VDR (1)			
hsa-miR-211	KCNMA1 (3)					
hsa-miR-302c	ESR1 (2)					
hsa-miR-326	PKM2 (2)	SMO (1)	GLI1 (1)	NOTCH2 (1)		
hsa-miR-422a**	CYP8B1 (2)					
hsa-miR-519c	HIF1A (4)					
hsa-miR-519d	CDKN1A (4)	PPARA (3)				
hsa-miR-520c	APP (3)	CD44 (3)				
hsa-miR-520g	VEGFA (2)					
hsa-miR-520h	SMAD6 (3)	ABCG2 (2)	CDKN1A (2)	VEGFA (2)	ID1 (1)	ID3 (1)
hsa-miR-522	SOX2 (1)					
hsa-miR-342	BMP7 (1)	GEMIN4 (1)				
hsa-miR-429	ZFPM2 (6)	ZEB1 (6)	ERRFI1 (4)	ZEB2 (4)	WASF3 (3)	BAP1 (1)

*, **Denote that the expression level is significantly higher (2 SD higher) in at least one of the donors than the endogenous control in only the PB or pNK, respectively. Number in parenthesis denotes the number of databases which indicate that the gene is targeted by the miR, in addition to having been validated previously.

### *Ex vivo* expansion of pNK cells

**Figure 3:**
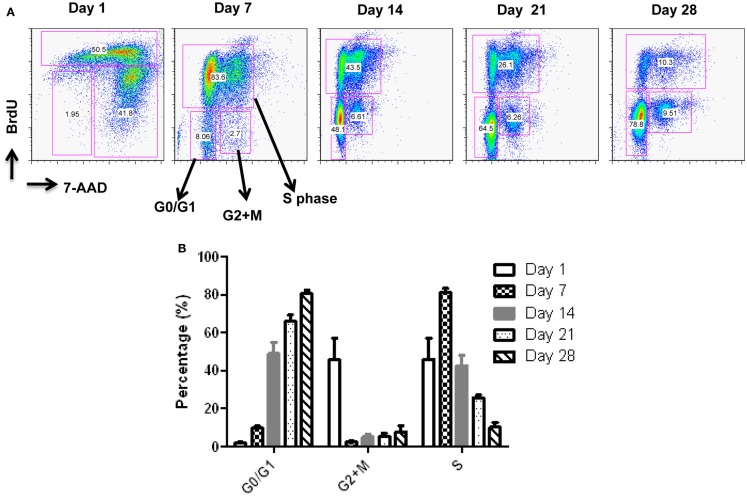
**Cell cycle analysis of expanded pNK cells**. **(A)** Representative APC-BrdU/7-AAD cell cycle analysis of *ex vivo*-expanded pNK at different time points as indicated. **(B)** Cell cycle analysis at different phases from *ex vivo*-expanded pNK at different time points as indicated (*n* = 5).

**Figure 4:**
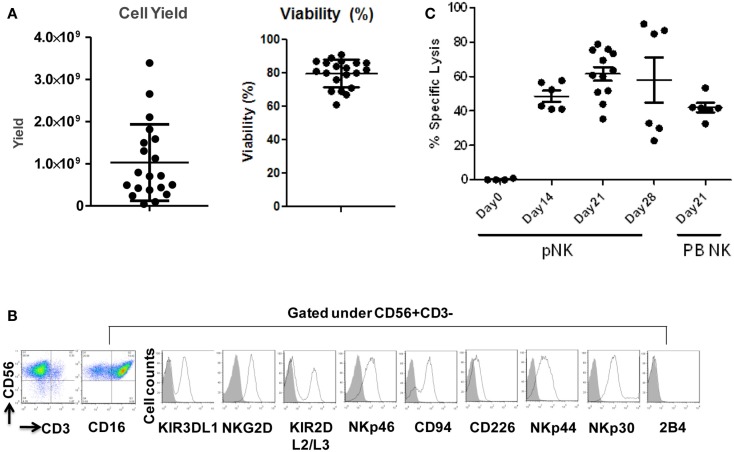
**Expansion, phenotype and functional characterization of *ex vivo*-expanded pNK cells**. **(A)** Cell yield and cell viability of Day 21 expanded pNK cells. **(B)** Phenotype characterization of Day 21 expanded pNK cells. **(C)** Cytotoxicity of expanded pNK cells against K562 at different time points as indicated in comparison with Day 21 expanded PB NK cells.

Starting from an average of 10 million pNK cells after isolation procedures, we attempted a series of optimizations of the pNK cell expansion process based on a previously described protocol for expansion of cytotoxic and helper T cells (Yssel et al., 1984). First, to optimize the feeder cell concentration, K562 cells and allogeneic PBMCs were tested at ratios of, 1:10, 1:5, and 1:1 (K562: PBMC), with the concentration of PBMC fixed at 1 × 10^6^/ml. As shown in Table 6, the ratio of 1:1 (1 × 10^6^/ml K562:1 × 10^6^/ml PBMC) resulted in the greatest NK cell expansion of 98-fold (*n* = 14), compared to 32-fold (*n* = 3) for the 1:5 ratio and 53-fold (*n* = 9) for the 1:10 ratio. Therefore the 1:1 ratio was used for further pNK cultivation. Next, we tested whether replenishing with fresh feeder cells could further enhance NK expansion during the cultivation process. To determine the optimal time window for replenishing with fresh feeder cells, cell growth kinetics of pNK cells at Day 7, 14, 21, and 28 were evaluated with BrdU/7-AAD double-staining followed by flow cytometry. As seen in Figure 3, at Day 7, the majority of cultured NK cells were in S-phase, indicating that the cells were proliferating. The percentage of actively proliferating/dividing cells decreased substantially during subsequent culture, suggesting Day 7 was the optimal window for re-stimulation. As shown in the Table 7, addition of fresh K562 and PBMC feeder cells at Day 7 resulted in a threefold increase in expansion of NK cells. This optimized 21-day NK culture method was repeated in 20 expansion experiments, yielding an average of 1.2 × 10^9^ CD56+CD3− NK cells with around 80% viability (Figure 4A).

**Table 6:** **Optimization of ratio of feeder cells: K562 to PBMC**.

Feeder Ratio (K562:PBMC)	Donors (*n*)	Fold expansion* range (min, max)	Average fold expansion
1:10	9	6, 131	53
1:5	3	11, 54	32
1:1	14	15, 358	98

*Fold expansion = absolute number of CD56+ CD3− NK cells on Day 21/absolute number of NK cells on Day 0.

**Table 7:** **Effect of feeder replenishing at different time points**.

Day of stimulation	Donors (*n*)	Fold expansion range (min, max)	Average fold expansion
Day 0	10	17, 83	41
Day 0 + Day 7	10	48, 395	148

### Characterization of *ex vivo*-expanded pNK cells

Immunophenotypic and miRNA changes were characterized in expanded pNK cells from 12 Combo units in comparison to unexpanded cells. First, Day 21 pNK cells showed a significant increase in the expression of activating receptors such as NKG2D, NKp46, NKp44, and NKp30, and a significant decrease in the expression of bidirectional receptor 2B4. The expression of inhibitory KIRs, including KIR3DL1 and KIR2DL2/L3 was similar for expanded and unexpanded cells (Figure 4B; Table 8).

**Table 8:** **Sub-population comparison of Day 21 expanded pNK versus unexpanded pNK cells**.

NK Sub-populations	Day 21 pNK (*n* = 12 U)		Day 0 pNK (*n* = 16 U)		*p* Value
	Mean (%)	SD	Mean (%)	SD	
CD56+CD16−	35.98	18.18	60.94	16.58	**
CD56+CD16+	63.80	18.32	39.05	16.58	**
KIR3DL1+	17.92	13.44	12.31	8.11	NS
KIR2DL2/L3+	21.10	10.48	21.89	8.65	NS
NKG2D+	89.28	12.88	42.11	17.79	***
NKp46+	88.74	5.34	6.98	4.33	***
CD226+	18.79	12.14	15.97	6.66	NS
NKp44+	64.13	16.65	9.48	5.27	**
NKp30+	84.53	12.40	39.08	19.06	***
2B4+	0.89	0.99	11.07	5.90	***
CD94+	74.82	12.45	71.31	13.94	NS

*p < 0.05; **p < 0.01; ***p < 0.001.

We have also investigated the immunophenotype of PB NK expanded using the optimized isolation and expansion procedure established for pNK in nine donors. Comparison studies of expanded PB NK cells to expanded pNK cells have revealed that the most profound difference was the lower expression of NKp44 (Table 9). Less cytotoxic activity was also observed with expanded PB NK cells at Day 21 (42 ± 7%) (Figure 4C).

**Table 9:** **Sub-populations comparison in Day 21 expanded pNK versus Day 21 expanded PB NK**.

NK sub-populations	Combo (12 U)		PB (9 U)		*p* Value
	Mean (%)	SD	Mean (%)	SD	
CD16−	35.98	18.18	28.39	7.78	NS
CD16+	63.80	18.32	71.50	7.78	NS
KIR3DL1+	17.92	13.44	8.83	6.44	NS
KIR2DL2/L3+	21.10	10.48	37.40	17.82	*
NKG2D+	89.28	12.88	73.27	20.06	NS
NKp46+	88.74	5.34	64.97	17.26	**
CD226+	18.79	12.14	4.80	1.72	NS
NKp44+	64.13	16.65	16.00	3.82	**
NKp30+	84.53	12.40	55.77	1.96	*
2B4+	0.89	0.99	2.16	1.76	NS
CD94+	74.82	12.45	71.76	20.35	NS

*p < 0.05; **p < 0.01; ***p < 0.001.

Moreover, Expression of 23 miRNAs was increased while 31 other miRNAs were downregulated after *ex vivo* expansion (Table 10). Interestingly, one of the miRNAs found to be upregulated was has-miR-155, which when overexpressed has been shown to increase NK cell function via enhanced induction of IFN-γ (Trotta et al., 2012).

**Table 10:** **Differentially regulated miRNAs during pNK *ex vivo* expansion**.

miRNA	Fold change in Day 21 versus Day 0	*p* Value
hsa-miR-520g	−2820.57	6.33E−05
hsa-miR-520h	−2803.02	4.35E−04
hsa-miR-518a	−2300.60	5.72E−05
hsa-miR-517b	−1669.82	8.11E−05
hsa-miR-451	−1626.65	2.40E−03
hsa-miR-518c	−609.88	2.93E−04
hsa-miR-127	−557.55	3.40E−04
hsa-miR-517a	−288.89	8.27E−05
hsa-miR-382	−273.34	1.77E−04
hsa-miR-519d	−245.09	7.13E−04
hsa-miR-486	−149.95	6.29E−03
hsa-miR-518b	−112.10	9.70E−05
hsa-miR-522	−85.30	2.58E−03
hsa-miR-376a	−72.14	2.95E−03
hsa-miR-198	−70.93	1.13E−03
hsa-miR-126	−51.46	1.97E−04
hsa-miR-487b	−47.68	3.32E−03
hsa-miR-519c	−47.52	4.88E−03
hsa-miR-518e	−34.89	5.06E−03
hsa-miR-433	−18.07	5.68E−04
hsa-miR-125b	−16.66	4.66E−04
hsa-miR-214	−16.38	5.74E−03
hsa-miR-130a	−12.98	4.13E−03
hsa-miR-518d	−10.75	8.73E−03
hsa-miR-99a	−7.91	2.03E−03
hsa-miR-515-3p	−4.93	2.50E−03
hsa-miR-95	−4.73	9.30E−04
hsa-miR-30a-3p	−3.06	1.20E−03
hsa-miR-30d	−2.59	4.32E−03
hsa-miR-26a	−1.75	7.81E−03
hsa-miR-191	−1.32	2.12E−03
hsa-miR-331	1.53	6.74E−03
hsa-miR-181c	1.71	1.24E−03
hsa-miR-142-3p	2.24	5.55E−03
hsa-miR-155	2.48	3.47E−03
hsa-miR-24	2.66	1.03E−03
hsa-miR-23a	3.07	8.50E−03
hsa-miR-142-5p	3.21	3.34E−04
hsa-let-7d	3.22	3.29E−03
hsa-miR-195	3.77	2.92E−04
hsa-miR-141	3.80	5.20E−03
hsa-miR-98	3.83	2.96E−03
hsa-miR-222	3.86	7.71E−03
hsa-miR-545	5.28	5.60E−03
hsa-miR-642	7.17	2.82E−04
hsa-miR-21	13.46	1.94E−04
hsa-miR-210	13.87	6.66E−03
hsa-miR-221	21.73	3.04E−03
hsa-miR-34c	45.37	5.26E−04
hsa-miR-135b	49.26	1.17E−03
hsa-miR-34a	66.38	8.08E−03
hsa-miR-10a	72.27	1.27E−03
hsa-miR-380-3p	921.50	1.18E−03
hsa-miR-520a	10892.33	2.38E−05

The cytolytic activity of expanded pNK was evaluated in a FACS-based PKH26/TO-PRO-3 cytotoxicity assay against K562 cells. As shown in Figure 4C, while unexpanded pNK cells showed minimal cytolytic activity, there was a significant enhancement of cytotoxicity against K562 cells by pNK cells at Day 21 versus Day 14 at an E:T ratio of 10:1 (63 ± 15% versus 45 ± 4%, *p* < 0.001). The increase in cytolytic activity after 21-day expansion was associated with the increased expression in activating receptors (Table 8) and miRNAs (Table 10). Extended cultivation to 28 days did not result in further increases in activity.

### *In vitro* anti-tumor cytolytic activity of expanded pNK cells

**Figure 5:**
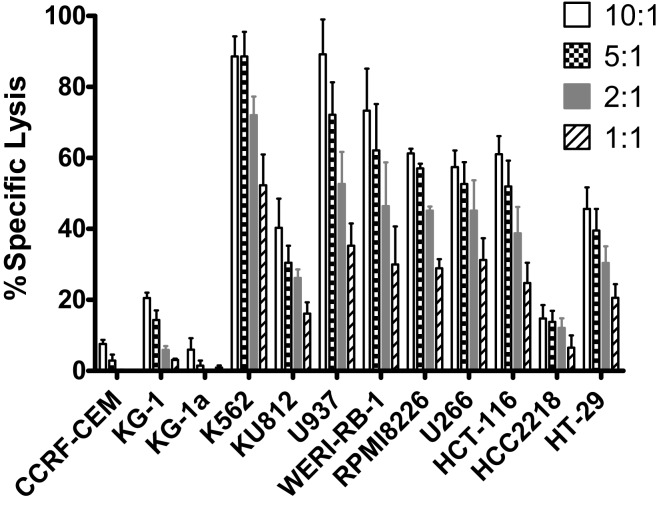
**Cytotoxicity of *ex vivo*-expanded Day 21 pNK cells against a wide range of tumor cell lines**. Cytotoxicity of *ex vivo*-expanded pNK cells (*n* = 6) against a wide range of tumor cell lines at E:T ratio of 10:1, 5:1, 2:1, and 1:1 as indicated.

To further evaluate expanded pNK cell activity against a range of tumor types, 11 additional tumor cells lines were co-cultured with Day 21 expanded pNK cells, and NK cell cytolytic activity was measured at E:T ratios of 10:1, 5:1, 2:1, and 1:1 in a 4-h LDH release assay. At an E:T ratio of 10:1, expanded pNK cells exhibited greater than 50% cytotoxicity against multiple tumor cell lines, including U937 (89.2 ± 9.8%), WERI-RB-1 (73.3 ± 11.8%), RPMI8226 (61.3% ± 1.3%), HCT-116 (61 ± 5.1%), U266 (57.4 ± 4.7%), as well as and K562 cells (88.6 ± 5.6%) (Figure 5). Cytolytic activity of expanded pNK cells against tumor lines was dose-dependent. Taken together, these results demonstrated that expanded pNKs have the ability to kill a wide variety of tumor cells derived from leukemia and solid tumors.

## Discussion

We have herein described, for the first time, that HPDSC (a product being developed by CCT for hematopoietic reconstitution in hematologic diseases requiring transplant) combined with donor-matched UCB, are a novel and rich source of pNK cells. Using a highly robust isolation method, we have achieved large numbers pNK cells in our experiments (approximately 1.5 × 10^7^ cells) from single Combo unit with a high proportion of viable cells (>70%), of which approximately 71% of the population is composed of CD56+CD3− cells. To overcome the hurdle of limited cell number for NK immunotherapy, we established a 21-day feeder cell-based NK cultivation process. Recently, several groups have successfully shown robust NK cell proliferation using K562 cells expressing 4-1BB Ligand and membrane-bound IL-15 (mbIL-15) or 4-1BB Ligand and mbIL-21 for expansion (Lee et al., 2010; Denman et al., 2012; Lapteva et al., 2012). Our initial culture optimization utilizing unmodified K562 cells and allogeneic PBMC resulted in an average of 1.2 × 10^9^ cells per donor that were >80% CD56+CD3− and with good consistency across donors. It is conceivable that potential NK cells with higher fold expansion, longer telomere, and less senescence (Denman et al., 2012) can be achieved by switching to genetically modified K562 cells. In addition, Xing et al. (2010) reported that UCB derived NK cells have low cytolytic activity due to impaired lytic immunological synapse formation that can be enhanced by addition of IL-2 during *ex vivo* expansion. In our study, while uncultured pNK cells showed little cytolytic activity against K562 cells, this activity significantly increased after 21 days of *ex vivo* expansion, and was associated with increased expression of NKG2D, NKp46, NKp44, and several miRNAs.

In recent years, human placenta has emerged as a valuable source of several stem/progenitor cell populations of mesenchymal and hematopoietic origin with therapeutic potential. However, little information is available on the role of pNK cells for cellular immunotherapy. We report here that pNK cells are largely similar to UCB NK cells, both phenotypically and functionally. We can significantly increase the number of feed stock for generating an NK cell-based therapeutic agent by combining NK cells from donor-matched HPDSC and UCB. In attempts to compare gene expression profiles in pNK cells to PB NK cells, we discovered that individual donor variations in gene expression profiles were too large to evaluate differences between the placenta and PB NK sources. As an alternative strategy, we performed analysis of miRNA expression, which appeared to be more consistent across individual donors, to identify profiles that were unique to pNK or PB NK cells. We identified four unique and 20 highly expressed miRNAs in pNK cells, and eight unique and 29 highly expressed miRNAs in PB NK cells. Target gene prediction analysis returned 14 highly expressed miRNAs in pNK and 24 highly expressed miRNA in PB NK with more than one target gene. In PB NK, highly expressed miRNAs included has-let-7a, has-let-7g, has-mir-133b, has-mir-181b, and has-mir-181d that target the anti-apoptotic genes Bcl-2 and Bcl-2L, which may indicate that PB NK cells are closer to reaching the limit of cell expansion than pNK cells. Additionally, miRNA hsa-mir-146b is highly expressed in PB NK cells, and its target TRAF6 has been reported to down-regulate NF-kB activity, suppress cell proliferation and enhance chemosensitivity (Paik et al., 2011). TRAF6 plays a critical role in innate and adaptive immunity (Chiffoleau et al., 2003) in conjunction with genes such as MAPK14, IL6, and FOS, which are targeted by has-mir-24, has-mir-7a, and has-mir-222 respectively, all found in our study to be highly expressed in PB NK. Furthermore, we identified a has-mir-181 group (has-mir-181b, has-mir-181c, has-mir-181d) that was threefold higher in PB NK. These miRNAs target nemo-like kinase, a regulator of Notch signaling, which plays an important role in the development of NK cells from CD34+ HSCs and IFN-γ production in primary CD56+ NK cells (Cichocki et al., 2011). Lastly, within the group of highly expressed miRNAs in pNK, we have identified miRNA: mRNA target pairs comprised of pluripotency markers in stem cells and cell cycle regulators, such as SOX2, BMPR1, SMO, AKT1, ATM, RAF1, and MTOR, most of which are not present in the targeted gene list (both experimental and validated) of miRNAs in PB NK. Furthermore, comparison studies have revealed the higher expression of NKp44 and greater cytotoxic activity against K562 at Day 21 (42 ± 7%) from expanded pNK compared to that of expanded PB NK cells.

In conclusion, we have characterized pNK cells from donor-matched HPDSC and UCB. pNK cells showed a distinct phenotype and miRNA profile from PB NK cells. We have demonstrated that pNK cells can be readily obtained from Combo units. These cells can be expanded, characterized, and activated to yield clinically relevant quantities of a highly cytotoxic cellular product with potential as a treatment for a wide range of hematological cancers. Taken together, the results presented here provide an important advance in the development of NK cell-based therapeutic products.

## Conflict of Interest Statement

Lin Kang, Vanessa Voskinarian-Berse, Eric Law, Tiffany Reddin, Mohit Bhatia, Yuhong Ning, Wolfgang Hofgartner, Stewart Abbot, Xiaokui Zhang, and Robert Hariri hold Celgene employment, equity ownership, and patents; David Dong, Timothy Maguire, and Martin Yarmush have received research funding from Celgene.
